# Technical efficiency of sub-district level hospitals in Bangladesh: a comparative frontier analysis

**DOI:** 10.1186/s13561-026-00748-6

**Published:** 2026-03-09

**Authors:** Md. Zahid Hasan, Edward JD Webb, Silviya Nikolova, Khadija Islam Tisha, Zahidul Quayyum, Tim Ensor

**Affiliations:** 1 Nuffield Centre for International Health and Development, University of Leeds, Leeds, LS2 9NL UK; 2 Academic Unit of Health Economics, Leeds Institute of Health Sciences, University of Leeds, Leeds, LS2 9NL UK; 3 Health Economics and Financing, Health Systems and Population Studies Division, icddr,B, Dhaka, 1212 Bangladesh; 4 Health Economics Outcomes Research Ltd, Rhymney House, Unit A, Copse Walk, Cardiff Gate Business Park, Cardiff, Cf23 8RB UK; 5 BRAC James P Grant School of Public Health, BRAC University, Dhaka, 1213 Bangladesh

**Keywords:** Technical efficiency, Stochastic frontier analysis, Primary healthcare, Sub-district hospitals, Data envelopment analysis, Bangladesh

## Abstract

**Background:**

In Bangladesh, sub-district hospitals (SDHs) are the first referral point for inpatient primary healthcare (PHC) services of the public providers in both rural and municipal corporation areas. These facilities also provide both outpatient and emergency healthcare services to the population at a minimum user fee. The efficient use of resources in primary-level healthcare facilities is essential for delivering quality healthcare services. Therefore, our aim was to estimate the technical efficiency (TE) of the SDHs in Bangladesh.

**Methods:**

We used an output-oriented data envelopment analysis (DEA) method to estimate the variable returns to scale (VRS) and constant returns to scale (CRS) TE of a total of 423 SDHs using data from the Local Health Bulletin -2017. To measure TE, we used workforce and inpatient beds as inputs and the number of inpatients and outpatients served by the hospitals in a month as output. We applied the Simar and Wilson model to find how the other internal and external characteristics of these hospitals influenced estimated TE score. We compared our DEA results with stochastic frontier analysis (SFA) and performed sensitivity analysis.

**Results:**

The average VRS and CRS TE of the SDHs were estimated to be 58.9% and 53.4%, respectively. Of the 423 SDHs, 15 were fully efficient in CRS, 30 were in VRS and 60 were scale efficient, while the rest operated below the efficiency frontier. The population density per bed, ratios of bed occupancy, ratios of beds to physicians, ratios of physicians to nurses, and administrative division had a significant positive influence, while lengths of stay and ratios of beds to nurses had a significant negative influence on the SDHs efficiency scores. The mean TE demonstrated that the SDHs, on an average, could improve their output by 42% using the existing level of input mix. The results were consistent in the sensitivity analysis.

**Conclusions:**

The average TE of the SDHs was half of the best score, suggesting there is scope for overall improvement among the inefficient SDHs by learning from the efficient SDHs. The Ministry of Health and Family Welfare (MOHFW) of Bangladesh allocates resources to SDHs based on the number of beds rather than based on an assessment of needs. The MOHFW could improve its monitoring system to investigate why some facilities are performing well using similar resources while others do not and adjust the allocation system to take into account the quantity and quality of care.

**Supplementary Information:**

The online version contains supplementary material available at 10.1186/s13561-026-00748-6.

## Background

Bangladesh is one of the most densely populated low-and-middle-income countries (LMICs) in the South Asian Region [1]. The country had a population of 165 million in 2022 [2]. By 2030, this population size is expected to increase to around 218 million [3]. The country has a pluralistic healthcare system with four key actors who are involved in providing healthcare services and are responsible for the functioning of the healthcare system. These actors include the government or public sector, the private for-profit sector, the private not-for-profit (NGO) sector, and donor agencies [4]. By the constitution, the public sector is responsible for providing basic healthcare services to every citizen [5]. The Ministry of Health and Family Welfare (MOHFW) through the Directorate General of Health Services (DGHS) and Directorate General of Family Planning (DGFP) manages a dual system of health and family planning services through a tier structure of healthcare delivery. The public healthcare system includes primary, secondary, and tertiary level healthcare facilities across the country. At the primary level, the public provider operates a total of 15,910 facilities in eight divisions of the country, including 424 sub-district level hospitals (SDH) or Upazila health complexes, 1,312 Union sub-centres, 87 Union health and family welfare centres, and 13,907 community clinics [6].

SDHs in Bangladesh are the first referral point for inpatient primary healthcare (PHC) services of public providers, both in rural and municipal corporation areas in Bangladesh. More than half of the SDHs are in the municipal area of Bangladesh. These facilities provide outpatient, emergency, and inpatient healthcare services to the population at a minimum user fee determined by the government. The sizes of these facilities currently ranges from a minimum of 10 to a maximum of 100 beds, with a total of 19,675 beds in 424 health facilities [6]. The majority of the facilities have 50 beds (345), followed by 31 beds (65), 10 beds (11), and 100 beds (3). SDHs also play an important role in operating the lower tier of the PHC system, e.g., union and community level health services. In 2018, SDHs served around 49.6% of total outpatients and 37.6% of total inpatients who visited public health facilities [6]. The latest Bangladesh National Health Account of 2020 estimated that SDHs and below-level facilities (e.g., union subcentres, community clinics) account for the highest share of public spending, amounting to 30.0% of the total public hospitals’ expenditure [7]. Resources have been allocated to SDHs primarily based on inpatient facilities (e.g., bed numbers) and staffing levels, resulting in significant disparities in per capita allocations of resources. Additionally, these differences do not appear to be explained by differences in healthcare needs across the areas [8]. While efficiency may not be explicitly articulated as the primary priority for the MOHFW, it plays a pivotal role in optimizing resource utilization, ensuring cost-effectiveness, and achieving greater healthcare outcomes considering limited resources [9]. One of the important aspects of high-quality health systems is that they are efficient in service delivery [10]. In Bangladesh’s Healthcare Financing Strategy 2012–2032, the optimization of healthcare management efficiency has been accorded the utmost priority. The importance of utilizing organizational and operational effectiveness in the healthcare system while concurrently developing the professional competency of the healthcare workforce is highlighted in this crucial policy framework [11]. The efficiency of health care providers can ensure the utilization of scarce resources to produce the best possible outputs. It can play a significant role in increasing service coverage and meeting the health needs of the population. Emphasizing efficiency to maximize output using the current level of inputs or minimizing costs to provide the current level of output can be a strategic approach aligned with the broader goals of the MOHFW in delivering effective healthcare services.

Technical efficiency (TE) is crucial for strengthening the PHC system of Bangladesh to optimise the use of limited available resources. Increased TE improves service delivery and quality of care [12], thus, enabling the health system to move towards universal health coverage by 2030 as part of the Sustainable Development Goal (SDG) 3.8 [13]. Additionally, measuring efficiency supports the evaluation of facility performance and data-driven decision-making to inform the adoption of best practices. A number of studies have been conducted in different countries to assess the technical efficiency of PHC providers using data envelopment analysis (DEA) [14–17]. DEA is a non-parametric method that is used to evaluate the TE of decision-making units (DMUs) by comparing their input–output ratios relative to an efficient frontier. One study also focused on the TE of public district hospitals in Bangladesh using a DEA approach [18]. However, no studies have been conducted to measure the efficiency of SDHs in Bangladesh. We aim to estimate the TE of selected SDHs utilizing publicly available secondary data.

## Methods

For estimating the TE score of the SDHs, we primarily used DEA, given that it can handle multiple inputs and multiple outputs. However, as the DEA does not account for random variation in data, we also examined the TE using stochastic frontier analysis (SFA) and compared the results. SFA accounts for random variation and inefficiency in the production process, but unlike DEA, it cannot directly handle multiple outputs, which is a main feature of production units like SDHs. Thus, we have estimated the TE of SDHs using DEA as our primary analysis, and in a supplementary analysis, compared the findings with various specifications of SFA.

### Theoretical framework

The theoretical framework for DEA is rooted in the concept of TE, derived from the theory of production. This theory assumes the existence of a mathematical function—referred to as the production function—that quantifies the relationship between inputs and outputs in a production process [19]. The production function defines the maximum output achievable from a given set of inputs, or alternatively, the minimum inputs required to produce a certain level of output. DEA uses this concept to evaluate the TE of DMUs [20]. It provides an approximation of the DMUs' efficiency, particularly in situations where there are several inputs and outputs, and efficiency is defined as:

$$\mathrm{Efficiency}= \frac{\text{Weighted sum of outputs}}{\text{Weighted sum of intputs}} \le 1$$

**Fig. 1:**
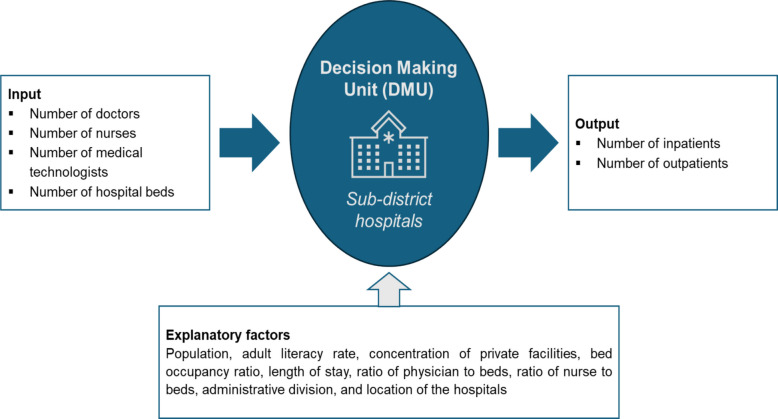
Components and variables for the technical efficiency analysis of SDHs

Based on multiple inputs and outputs, DEA identifies a frontier similar to a production frontier on which the relative performance of all DMUs in the sample can be compared [21]. DMUs are considered to be technically efficient among their peers if they maximise output from a given set of inputs. A DMU is fully (100%) efficient based on the available evidence if, and only if, there is no indication that any of its inputs or outputs can be improved without negatively impacting other inputs or outputs when compared to the performance of other DMUs [22]. By assumption, the efficient DMUs, being on the efficient frontier, will have an efficiency score of 1, and the inefficient DMUs, below the efficient frontier, will have an efficiency score of less than 1. It is noted that TE is a relative measure within the sample. Although DMUs on the efficient frontier are granted a 100% efficiency score, it is likely that they could further improve their productivity if compared with more efficient peers in a separate sample. Figure 1 represents the basic relationship of inputs, outputs, and explanatory factors of SDHs in Bangladesh. SDHs are the DMUs for producing healthcare services for the population in their regions. Details about the selection of variables and DEA model are discussed under the selection of input and output variables for efficiency analysis.

This efficiency analysis was conducted in two stages using secondary cross-sectional data from different sources (Table 1). In the first stage, the technical efficiency scores of the facilities were estimated by using an output-oriented DEA model. In the second stage, the associations of internal and external characteristics of the facilities with the TE score were identified by using the Simar and Wilson double bootstrap procedure (explained later) [27].

**Table 1:** Selected variables and sources of information

Variables	Indicators	Source of information
Input variables		
Number of beds	Total number of available beds	Local Health bulletin 2017 [23]
Doctors	Total number of doctors (specialists and primary care physicians)	Local Health bulletin 2017 [23]
Nurses	Total number of nurses available	Local Health bulletin 2017 [23]
Medical technologists	Total number of medical technologists available	Local Health bulletin 2017 [23]
Output variables		
Outpatient visits	Total number of outpatient visits	Local Health bulletin 2017 [23]
Inpatient admissions	Total number of inpatient days	Local Health bulletin 2017 [23]
Explanatory variables		
Population density per bed	Number of catchment populations in the sub-district per bed	Bangladesh Bureau of Statistics [24]
Bed occupancy ratio	Proportion of beds occupied over a specific year	Local Health bulletin 2017 [23]
Average length of stay	Average days a patient stayed at a hospital	Local Health bulletin 2017 [23]
Ratio of beds to physicians	Total number of beds per physician	Local Health bulletin 2017 [23]
Ratio of beds to nurses	Total number of beds per nurse	Local Health bulletin 2017 [23]
Ratio of nurses to physician	Total number of nurses per physician	Local Health bulletin 2017 [23]
Number of private facilities	Total number of registered private health facilities	Directorate General of Health Services [25]
Adult literacy rate	Percentage of the literate adult population	Sample Vital Statistics [26]

### Data sources

Data on the input and output variables for the DEA model (first stage of analysis) were extracted from the publicly available Bangladesh Local Health Bulletin 2017 [23] for SDHs. The bulletin consists of hospital services and monitoring statistics which are updated monthly using the online Management Information System (MIS) under the DGHS, MOHFW, Bangladesh. For the second stage analysis, data were extracted from multiple publicly available sources: hospital-related information from Local Health Bulletin 2017, sub-district-wise population data from the Bangladesh Bureau of Statistics (BBS) [24], the number of private facilities in a sub-district from the hospitals and clinic division of DGHS [25], and district-level adult literacy rate from Bangladesh Sample Vital Statistics as a proxy in the absence of such sub-district level statistics [26]. According to the Bangladesh Health Bulletin 2019, there are a total of 424 SDHs across the country [28]. However, in the local health bulletin, two facilities were listed under the SDHs category in Ullapara sub-district: 1) Ullapara SDH and 2) Ullapara 30-bed Sadar hospital. However, since the Ullapara 30-bed Sadar hospital is a different type of facility and we did not find its statistics in the local health bulletin, we have excluded it from our analysis.

### Analytical approach for efficiency measurement

Based on Farrell’s concept, several DEA models have been developed to analyse the TE of production units [29]. Two models are most commonly used for estimating efficiency frontiers. The first model, developed by Charnes, Cooper, and Rhodes (CCR) [30], assumes the efficiency frontier has a constant slope, meaning production follows constant returns to scale (CRS). Under this assumption, any level of increase in inputs will proportionately increase the level of outputs. CRS may be observed in contexts where machines or automated processes play a significant role, so that production can be doubled by doubling the levels of inputs. The second model, developed by Banker, Charnes, and Cooper (BCC) [31], assumes that production exhibits variable returns to scale (VRS), which means any increase in outputs is not proportionate to the increase in inputs. In the VRS assumption, an SDH may see a more than proportionate increase in output, or increasing returns to scale (IRS), or a less than proportionate increase in output, or decreasing returns to scale (DRS), or CRS. As the health service production process is mostly operated based on human factors, the VRS TE assumption of the BCC model is more appropriate, as it reflects the real-world complexities of service production. The VRS assumption does not follow a strict binding of optimal scale like CRS assumption does. This means the VRS efficiency only evaluates whether a production unit is efficient at its current scale of operation. Thus, any change in measured TE can be attributed to the presence of scale inefficiency, which is represented by the ratio of the scores from CRS TE and VRS TE [32]. Due to the relaxed assumption of VRS, the VRS TE score is always higher than or equal to the CRS TE. Scale efficiency determines whether an SDH is operating in optimal size or not. A scale efficiency score of 1 indicates that CRS TE and VRS TE scores are equal and the SDH is operating at optimal size. In our analysis, we have used both CRS TE based on the CCR model and VRS TE based on the BCC model to estimate the efficiency score and make a comparison between them. Previous studies have utilized both CRS and VRS assumptions in efficiency analysis as reported in systematic reviews [33, 34].

### Output-oriented DEA model analysing TE of SDHs

The orientation of the efficiency models reflects the appropriate direction of optimisation, and therefore it is important to specify. It is defined based on whether the DMUs have better control over inputs or outputs. Several studies have suggested that hospitals have relatively limited control over their outputs (e.g., the number of admissions) but greater control over their inputs (e.g., supply of instruments), thus an input-oriented approach might be used so that a desired level of output can be reached by using a minimal quantity of inputs [33, 34]. In some cases, organisations are allocated a fixed quantity of resources, and in such circumstances, an input-oriented model may be appropriate. Given the healthcare settings in Bangladesh, where there is an unmet need for healthcare services and hospitals have limited scope to adjust their inputs, we have utilized an output-oriented DEA model to estimate the efficiency score of the SDHs. The assumption is that SDHs maximize their outputs through efficient utilization of their current level of inputs. The demand for quality healthcare services tends to increase with population size, which necessitates increasing the outputs of public facilities to reduce the financial burden and increase people’s access to healthcare services. Moreover, in the context of Bangladesh, SDHs are allocated resources based on the number of hospital beds, rather than their service delivery. Thus, an output-oriented model is more reasonable for assessing the TE of SDHs aiming to improve outputs as much as possible given the fixed inputs. The linear programming version of the output-oriented BCC model is specified as follows [31]:

$$\mathrm{Max}\;\mathrm\phi\;=\;\sum_{\mathrm i=1}^{\mathrm s}\;{\mathrm A}_{\mathrm r}\;{\mathrm Y}_{\mathrm{ro}}\;+\;{\mathrm A}_0$$

$$Subject\,to$$

1 $$\sum_{i=1}^{m}{B}_{i} {X}_{{i}_{0}}=1$$

$$\sum_{r=1}^{s}{A}_{r}{Y}_{rj}+{A}_{0}-\sum_{i=1}^{m}{B}_{i} {X}_{ij}\le 0, \forall j=1,\dots ,n$$

$${A}_{r}{, B}_{i} \ge 0;{A}_{0} is free in sign$$

where, Y_rj_ = amount of output r from facility j, A_r_ = weight given to output r and $\sum_{r=1}^{s}{A}_{r}{Y}_{rj}$ is the weighted sum of outputs for facility j.

X_ij_ = amount of input i to facility j, B_i_ = weight given to input i, and $\sum_{i=1}^{m}{B}_{i} {X}_{ij}$ is the weighted sum of inputs for facility j.

*n* = number of facilities.

s = number of outputs.

m = number of inputs.

A_0_ > 0 defines increasing returns to scale, A_0_ = 0 defines CRS, and A_0_ < 0 defines decreasing returns to scale. The TE score is defined by ${\Phi }_{j}$ and it ranges between 0.00 and 1.00. If it is equal to 1.00, then the production from the SDH is efficient; while if it is less than 1.00, the SDH is inefficient.

### Selection of input and output variables for efficiency analysis

Health facilities transform its resources/inputs into a range of activities that ultimately contribute to the overall health status of the population [35]. There are two approaches used to measure the output of a healthcare facility. The first one is the process approach, which means that the output of a healthcare facility consists of intermediate activities/services such as the number of tests performed or patients served or patient days, etc., and the second one is the outcome approach which considers health status as the ultimate outcome of health services [36]. However, organisations rarely collect routine information about what health outcomes they produce [35]. In this study, we have followed the process approach as the SDHs do not have information on the health status of the population. Based on the process approach, the number of outpatient and inpatient visits at SDHs was used as the output variable of interest. In SDHs, statistics on both outpatient and inpatient visits for PHC services are available.

The selection of variables for the efficiency analysis was guided by literature and the availability of information in the current dataset [18, 23, 37]. Considering the production process in a healthcare facility, i.e., transforming the resources or inputs to services or outputs, the inputs are broadly classified as labour and capital. The labour input can be disaggregated into various professional groups, such as physicians/doctors, nurses, and administrative staff. In this analysis, we have included doctors, nurses, and medical technologists as labour inputs. We have used the number of beds in each facility as a proxy for capital input. The number of beds has been used for capital inputs mainly for two reasons. Firstly, the number of beds has been used as a proxy for capital inputs in many earlier studies [18, 38, 39]. Secondly, alternative measures such as the area of the facility buildings or the number of rooms, which could have been used to approximate the capital input in the DEA model were absent from the dataset.

### Second stage of DEA analysis: regression

The efficiency scores estimated from the DEA model were further analysed via regressing them against some selected external and internal characteristics of the SDHs to examine how these factors could affect the TE. The efficiency scores are bounded between 0 and 1. Estimating a model with a bounded dependent variable using ordinary least squares (OLS) can result in biased and inconsistent estimates in parametric models. This is because OLS does not account for the constraints imposed by the dependent variable’s limits. Additionally, since DEA scores are relative measures of efficiency and there is interdependence among the scores of SDHs, OLS regression is inappropriate and invalid for analyzing these scores [27, 40]. To overcome this issue, traditionally, Tobit models have been used to evaluate the factors affecting efficiency [41, 40]. Some studies have also used afractional regression model considering the outcome of interest is measured as a fraction, i.e., taking values between 0 and 1 [42].

However, Simar and Wilson have argued that the conventional approaches to statistical inference are invalid for DEA models due to serial correlation among the estimated efficiencies, and recommended using single and double bootstrap procedures based on a truncated regression model [27]. The rationale of bootstrapping is to approximate the true sampling distribution by replicating the data generation process. As the efficiency scores range from 0 to 1, the boundary estimation framework of DEA suffers from finite-sample bias, and the efficiency score is biased toward the value of 1. In this study, we followed the procedures applied by Simar and Wilson’s (2007) Algorithm 2 (see Appendix 1). In the case of the output-oriented DEA model, the Simar and Wilson method generates bias-corrected inefficiency scores following the Farrell distance measure. The user-developed commands ‘simarwilson’ by Badunenko and Mozharovskyi were used in Stata version 17 for the analysis with 100 replications for bias correction and 1000 replications for bootstrapping standard errors [43]. For the convenience of analysis and explanation, we inverted the inefficiency score to an efficiency score by using the ‘invert’ option [44].

#### Selection of internal and external factors

The explanatory variables for the second stage analysis were selected based on literature review on efficiency analysis and data availability in the context of Bangladesh [45–47]. Factors that may affect the efficiency of SDHs were classified into external factors, specifically density of catchment population per bed, administrative location of the facility, adult literacy rate of the region, concentration of private health facilities, and internal factors, specifically average length of stay (ALoS), bed occupancy ratio (BOR), ratio of beds to physicians (BTP), ratio of beds to nurses (BTN), and ratio of nurse to physician (NTP) (Table 1). We could not include variables like poverty rates in the analysis due to the unavailability of such information at the sub-district level.

### Stochastic frontier model

SFA is a parametric statistical technique of frontier estimation that assumes that each firm potentially produces less output due to some degree of inefficiency. The deviation from the efficiency frontier may be influenced by external factors, the use of inadequate technology, or random shocks. The relationship between the inputs used and the output produced is assumed to be stochastic. Thus, the deviation from efficiency can incorporate both inefficiency and noise in the data [48]. However, unlike DEA models, SFA is limited to analysing a single output.

In the Cobb–Douglas production function, for the SDHs, the SFA model can be written as:

2 $$\mathrm{ln}\left({y}_{i}\right)={\upbeta }_{j}+{\sum }_{j=1}^{k}{{\upbeta }_{j}\mathit{lnx}}_{\mathit{ji}}+{v}_{i}-{u}_{i}$$

with

$${v}_{i}\sim {N(0,{\delta }^{2}}_{v})\ and\ {u}_{i}\sim {N}_{+}{(0,{\delta }^{2}}_{u})$$

 

Where $v$ represents the stochastic nature of the production process and possible.

measurement errors of the inputs and output, and the $$u$$ is the possible inefficiency of the firm. We assume that v and u are independent. If $$u=0$$ the firm is 100% efficient, and, if $$u>0$$, then there is some inefficiency. The number of inputs is expressed by $$j$$, $$i$$ is the SDH, $${y}_{i}$$ is the output of the $$i$$-th SDH, $${x}_{ji} $$ is the input $$j$$ of the $$i$$-th SDH, $$\upbeta$$ the parameters to be estimated, $${v}_{i}$$ is a systematic random error, to account for statistical noise with zero mean and unknown variance, and $${u}_{i}$$ the non-negative random variable associated with TE of SDH $$i$$.

The TE of SDH $i$ can be expressed as:

3 $${TE}_{i}=E[\mathrm{exp}(\raisebox{1ex}{$-{u}_{i}$}\!\left/ \!\raisebox{-1ex}{${v}_{i}-{u}_{i}$}\right.)]$$

While the Cobb–Douglas form is easy to estimate, its main drawback is that it assumes constant input elasticities and returns to scale for all SDHs. In contrast, the translog form does not impose these restrictions but is prone to issues such as degrees of freedom problems and multicollinearity [49, 50]. In this study, we estimated three models: the Cobb–Douglas form and the translog form, considering the single output of total patient days (patient days were estimated considering the outpatient visit as one day and multiplying the number of inpatients by the length of stay), and the multi-output distance function (Appendix 2). We have tested the log-likelihood, Akaike information criterion (AIC), and Bayesian information criterion (BIC) of the Cobb–Douglas model, SFA model with half-normal, exponential, gamma, and truncated distributions. Following the model fit parameters, the optimal model was considered based on lower AIC and BIC. We have conducted the SFA analysis by using the ‘sfcross’ command of STATA 17 [51]. The efficiency score was estimated based on Eq. (3). The command ‘bc’ under the ‘sfcross’ produced estimates of TE based on the estimator of Battese and Coelli 1988 [52].

### Sensitivity analysis

We conducted a one-way sensitivity analysis to assess the robustness of efficiency scores derived from different efficiency model specifications. The base output-oriented DEA model used inpatients and outpatients as outputs and doctors, nurses, medical technologists, and the number of beds as inputs. The sensitivity analysis involved several modifications of the base output-oriented model to derive efficiency scores: a) excluding facilities with an efficiency score of 1 generated in the main model, b) using an input-oriented DEA model, c) using total patient days as output in the SFA model, and d) using a multioutput distance function SFA model [53]. All analyses were conducted using Stata 17 and Microsoft Excel [30].

## Results

### Descriptive statistics

Table 2 presents the descriptive characteristics of the selected inputs and outputs for the DEA analysis. We found that on average there were 17 doctors including consultants and the number of doctors ranged from 3 to 36 across the SDHs. On average there were 24 nurses in an SDH and the number of nurses ranged from 2 to 36. The number of medical technologists ranged from 1 to 12 in the SDHs, and on average there were 6 medical technologists. The SDHs had 47 beds with a minimum of 10 to a maximum of 100. On average 452 inpatients and 4,740 outpatients were treated per month in the studied SDHs.

**Table 2:** Descriptive statistics of input and output variables of sub-district hospitals (*n* = 423)

Variables	Mean	Standard deviation	Minimum	Maximum
Inputs				
Doctors (including consultants)	17	6	3	36
Nurse	24	7	2	36
Medical technologist	6	2	1	12
Hospital bed	47	10	10	100
Outputs				
Inpatient admissions per month	452	237	4	1,389
Outpatients visit per month	4,740	2,276	300	13,640

### Efficiency estimates

Results from the DEA analysis showed that the SDHs were on average 53.4% technically efficient under the CRS assumption and 58.9% technically efficient under the VRS assumption (Table 3). However, the average scale efficiency of the SDHs was 90.7% which implied that the facilities on average were below their optimal size. Considering CRS TE, 15 (3.5%) SDHs were operating at a fully efficient level, however, with VRS TE, 30 (7.1%) SDHs were operating at a fully efficient level. While examining the mean efficiency scores by different characteristics of SDHs, we found that SDHs with up to 31 beds were on average 61.1% efficient under the CRS assumption, which was higher compared to the 50 or more bed SDHs (0.520), and the difference was statistically significant. Similarly, on average, these SDHs had higher VRS technical efficiency and scale efficiency compared to the 50 or more bed SDHs, and the differences were statistically significant. The average TE of the SDHs across geographic divisions (i.e., where the SDHs were located) varied significantly under both CRS and VRS assumptions, from the lowest in Barisal Division (0.437 vs 0.466) to the highest in Sylhet Division (0.694 vs 0.747). Average Scale efficiency was the highest among the SDHs in the Rangpur Division (0.956) and lowest in the Chattogram Division (0.873), and the difference in efficiency scores across the divisions was statistically significant. While examining the CRS and VRS TE of the SDHs by location, we found that on average, the SDHs had significantly higher TE in urban areas compared to the rural areas under both CRS (0.527 vs 0.494; *p* < 0.01) and VRS (0.599 vs 0.528; *p* < 0.05) assumptions.

**Table 3:** Summary of efficiency scores, average technical and scale efficiency scores by size and location of sub-district hospitals

Characteristics	Number of SDH	CRS technical efficiency	VRS technical efficiency	Scale efficiency
	***n***	***Mean (Standard error)***		
Total				
Mean efficiency score	423	0.534 ± 0.009	0.589 ± 0.010	0.907 ± 0.005
Minimum efficiency score	423	0.085	0.085	0.615
Maximum efficiency score	423	1.000	1.000	1.000
Fully efficient SDHs (efficiency score is 1)	423	15	30	60
Hospital size ^a)^		*p* < *0.005*	*p* < *0.001*	*p* < *0.005*
Up to 31 bedded	72	0.611 ± 0.030	0.688 ± 0.029	0.890 ± 0.022
50 bedded	348	0.520 ± 0.010	0.570 ± 0.011	0.913 ± 0.004
100 bedded	3	0.379 ± 0.063	0.579 ± 0.092	0.652 ± 0.027
Division ^a)^		*p* < *0.001*	*p* < *0.001*	*p* < *0.001*
Barisal	34	0.437 ± 0.034	0.466 ± 0.038	0.951 ± 0.017
Chattogram	90	0.516 ± 0.026	0.593 ± 0.026	0.873 ± 0.016
Dhaka	77	0.533 ± 0.019	0.613 ± 0.021	0.876 ± 0.011
Khulna	50	0.533 ± 0.025	0.570 ± 0.025	0.932 ± 0.009
Rajshahi	59	0.512 ± 0.023	0.564 ± 0.023	0.903 ± 0.009
Rangpur	49	0.569 ± 0.032	0.593 ± 0.031	0.956 ± 0.008
Sylhet	34	0.694 ± 0.034	0.747 ± 0.031	0.923 ± 0.021
Mymensingh	30	0.511 ± 0.030	0.562 ± 0.036	0.916 ± 0.016
Location ^b)^		*p* < *0.01*	*p* < *0.05*	*p* = *0.425*
Rural	190	0.494 ± 0.021	0.528 ± 0.022	0.94 ± 0.009
Urban	233	0.527 ± 0.016	0.599 ± 0.018	0.887 ± 0.009

*SDH *Sub-district hospitals, *CRS *Constant returns to scale, *VRS *Variable returns to scale

^a^^)^Kruskal Wallis test

^b^^)^Mann–Whitney U Tests

**Fig. 2:**
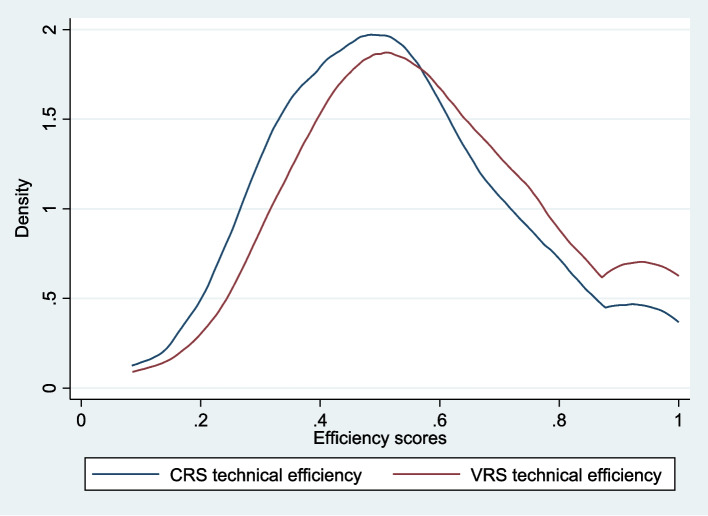
Density of technical efficiency scores under constant and variable returns to scale (*n* = 423). CRS = constant returns to scale; VRS = variable returns to scale

The density plot of the CRS and VRS efficiency scores is presented in Fig. 2. The majority of the SDHs were 40% to 60% technically efficient in both CRS and VRS assumptions. Less than one-fifth of the SDHs were 80% or more technically efficient in the VRS assumption. The density of VRS efficiency (red line) is slightly shifted to the right of the density of CRS efficiency (blue line), indicating that the VRS efficiency scores are generally higher.

### Findings from the second stage analysis

The results of the Simar and Wilson bias-corrected regression models are presented in Table 4. The positive coefficients indicated that factors would increase the efficiency and the negative coefficients indicated that factors would reduce the efficiency score. We found that the efficiency of hospitals increased significantly with population density per hospital bed. Hospitals in areas with more than 6,500 people per bed have a 0.0396 higher bias-corrected efficiency score on average compared to the reference group.

**Table 4:** Truncated regression using efficiency scores and bias-corrected efficiency scores of sub-district hospitals

Characteristics	Dependent variable = Bias corrected efficiency score ^a)^
Population density per hospital bed	
< = 4300 per bed	Ref
> 4300 to < 6500 per bed	0.0276 (−0.006,0.061)
> 6500 per bed	0.0396* (0.000,0.079)
Adult literacy rate in percentage	0.0015 (−0.000,0.003)
Number of private facilities in the area	0.0012 (−0.001,0.003)
Bed occupancy ratio (BOR)	0.0028*** (0.002,0.003)
Average length of stay (ALoS) in days	−0.0679*** (−0.083,−0.052)
Ratio of beds to physicians (BTP)	0.0180* (0.001,0.035)
Ratio of beds to nurses (BTN)	−0.0425** (−0.068,−0.017)
Ratio of physician to nurse (PTN)	0.1751*** (0.097,0.254)
Administrative division	
Barisal	Ref
Chattogram	0.0472 (−0.011,0.106)
Dhaka	0.1428*** (0.083,0.203)
Khulna	0.0784* (0.018,0.139)
Rajshahi	0.1231*** (0.062,0.184)
Rangpur	0.1053** (0.041,0.170)
Sylhet	0.1482*** (0.080,0.216)
Mymensingh	0.0911* (0.014,0.168)
Area	
Rural	Ref
Urban	0.0115 (−0.016,0.039)
Constant	0.0933 (−0.100,0.287)
Sigma	0.1226*** (0.114,0.131)
n	418.000
*Model significance*	***

^a)^ Simar and Wilson’s bootstrap DEA [27]; Ref = reference category for comparison

^*^p < 0.05

^**^p < 0.01

^***^p < 0.001

Bed occupancy ratio, ratio of beds to physicians, and ratio of physicians to nurses had significantly positive associations with efficiency, suggesting that changes in these factors could improve the TE of the SDHs. For example, on average, a one-unit increase in the ratio of beds to physicians results in a 0.0180 unit increase in the predicted bias-corrected technical efficiency score. On the other hand, the average length of stay and ratio of beds to nurses had a significant negative association with the efficiency score, meaning that a one unit change in these factors will reduce the efficiency by 0.067 and 0.042, respectively. Compared to the SDHs in the Barisal Division, the TE of SDHs in Dhaka, Khulna, Rajshahi, Rangpur, Sylhet, and Mymensingh were significantly higher. The percentage of adult literacy, the number of private facilities in the area, and the urban location of SDHs were positively associated with the efficiency score, however, these associations were not statistically significant.

### Comparison with the stochastic frontier analysis model

We have tested the SFA models assuming truncated normal distributions for the inefficiency term. Based on the likelihood ratio tests, AIC, and BIC we found that the simpler Cobb–Douglas model was not sufficient, and there is evidence to suggest that the more complex translog model is a significantly better fit for the data. The SFA model with a multioutput distance function also had a better fit as it had the highest log likelihood and lowest AIC (Supplementary Table 1).

Table 5 presents the pairwise correlations between the SFA and DEA efficiency scores of the SDHs. The results indicated strong positive correlations between SFA and DEA efficiency scores across the different models. While using multiple outputs, the correlation between the SFA translog distance function and DEA efficiency scores is 0.7215 under the CRS assumption and 0.7223 under the VRS assumption. Similarly, for total patient days as output, in both SFA and DEA models, the correlation was 0.7167 under CRS and 0.6761 under VRS. All associations were statistically significant (*p* < 0.001). The consistent pattern of efficiency estimates across different models indicated the robustness of the efficiency measurements.

**Table 5:** Pairwise correlations of stochastic frontier analysis and data envelopment analysis efficiency scores

Variables ^a)^	SFA_TDF	DEA_CRS	DEA_VRS	SFA_TPD	DEA_CRS	DEA_VRS
SFA_TDF ^b)^	1					
DEA_CRS ^c)^	0.7215	1				
DEA_VRS ^c)^	0.7223	0.9215	1			
SFA TPD ^d)^	0.8937	0.7167	0.6761	1		
DEA_CRS TPD ^d)^	0.7104	0.8473	0.8345	0.7455	1	
DEA_VRS TPD ^d)^	0.7304	0.7771	0.9105	0.707	0.8978	1

In all correlations: the level of significance was *p* < 0.001

*DEA *Data envelopment analysis, *CRS *Constant returns to scale, *VRS *Variable returns to scalea)

^b)^ Translog multioutput (inpatients and outpatients) distance function in stochastic frontier analysis

^c)^ Number of inpatients and outpatients were used as output

^d)^ Total patient days were output

### Findings from the sensitivity analysis

**Fig. 3:**
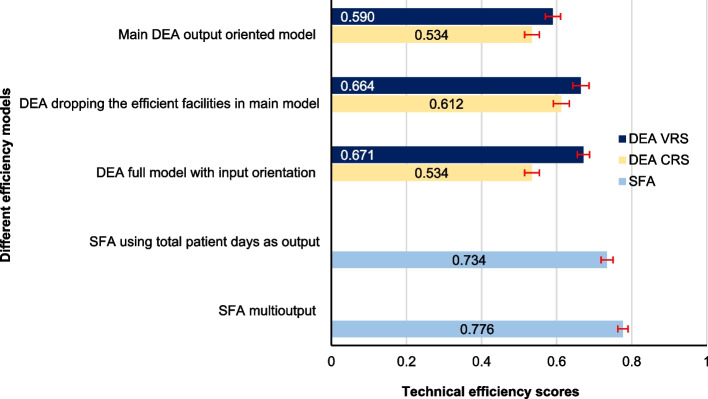
One-way sensitivity analysis of technical efficiency scores. DEA = Data Envelopment Analysis; SFA = Stochastic Frontier Analysis

Figure 3 presents the results of the sensitivity analysis, comparing the technical efficiency scores across various models. The average efficiency score of SDHs ranged from 0.534 to 0.776 in the different types of models. The main model had an average CRS efficiency score of 0.534 and a VRS efficiency score of 0.590, serving as a baseline for comparison. Excluding fully efficient SDHs (i.e., those that had an efficiency score of 1 in the main model) resulted in efficiency scores of 0.612 in CRS and 0.664 in VRS, on average. Using all variables in an input-oriented DEA model, the average CRS and VRS efficiency scores were 0.534 and 0.671, respectively. When using SFA with total patient days as the output, the efficiency score was 0.734, while in the multioutput translog distance function SFA, the efficiency score became 0.776.

## Discussion

The findings of this study indicated that SDHs, the key contact point for public primary inpatient healthcare in Bangladesh, were technically inefficient in terms of utilizing the facility inputs. The average VRS and CRS technical efficiency of the SDHs were approximately 58.9% and 53.4%, respectively, indicating that the resources were not being utilized optimally. Of the 423 SDHs, only 15 (3.5%) and 30 (7.1%) facilities were estimated to be technically efficient, depending on the approach used, and 60 (14.2%) SDHs were optimal in size. Moreover, the performance of some SDHs was observed to be very low (efficiency was less than 20%), which raised much concern for the health policymakers in Bangladesh. In resource-limited settings like Bangladesh, optimizing available resources is crucial. The presence of inefficiencies in healthcare systems might hinder the country’s progress to achieve key global health targets like SDGs, as well as the move towards universal health coverage by 2030 [13]. To address the challenges effectively, a renewed focus on optimizing existing healthcare resources is essential.

Previous studies on the efficiency of PHC facilities conducted using a DEA approach reported different findings in different settings. A study conducted in Ghana in 2017 showed that the average efficiency of public hospitals was 0.50, which was similar to the efficiency of SDHs in Bangladesh [54]. Another study conducted in 2022 on PHC facilities in Afghanistan showed that the average TE of the facilities was 0.74, which is higher compared to the efficiency of SDHs in Bangladesh [55].

The findings of this study suggest that if the SDHs could be operated on the efficiency frontier, they could, on average, provide services to at least 42.1% more patients, utilizing their current level of inputs. Technically efficient facilities had an average of 14 doctors, 16 nurses, 4 medical technologists, and 42 beds (results not shown), which was lower compared to the average number of 17 doctors, 24 nurses, 6 medical technologists, and 47 beds. However, most of the SDHs in Bangladesh are 50 bedded, and adjusting the health facility size (i.e., reducing the number of beds or shifting beds to other hospitals) requires changes in infrastructure levels, which is a long-term process. So, it may be more practical to focus on other inputs such as staffing levels, as this may be adjusted on a much shorter time horizon, considering the current efficiency level of the SDHs.

This study found that about 14.2% of the SDHs were scale-efficient, meaning they were operating at the optimal size. However, 61.7% of SDHs were operating at increasing returns to scale, thus, there is a possibility of expansion of outputs if the existing inputs are properly utilized [56]. The scale of the inefficient facilities can be adjusted based on the available inputs. Technically inefficient facilities could rather reduce their input mix and invest in other hospital characteristics that could help to increase the utilization of the facilities. The adjustments in the input mix can be informed by insights gained from the efficient SDHs.

We found that the efficiency of SDHs increased with an increase in population density per hospital bed. This could be explained by considering the current resource allocation policy for SDHs in Bangladesh. The SDHs get resources based on the number of beds in a facility rather than the population density of the catchment area, potentially leading to lower utilization of SDHs in areas with a lower population density. Similar findings were reported in a study from Chile, which revealed that increased population density in the catchment area of a PHC was linked to higher TE in regional health systems [57].

The Simar and Wilson's model showed that the bed occupancy ratio, average length of stay, ratio of physician to nurse, and ratio of beds to nurses were significant determinants of the SDHs’ efficiency score. For improving the efficiency of inefficient SDHs, feasible changes in input mix can have an important contribution to improving technical efficiency, such as increasing the ratio of beds to physician and ratio of physician to nurse, which implies that the number of physicians can be increased in the inefficient facilities to a certain level based on the demand and ratio of efficient SDHs to make them efficient. However, recent statistics showed that 25% of physician positions and 10% of nurse positions are vacant compared to what is allocated in public health facilities in Bangladesh [58]. Despite the high workload pressures faced by physicians and nurses at the SDHs in Bangladesh [59], such vacancies of human resources reduce efficiency and hinder the optimal utilization of available human resources. The efficiency score of SDHs was found to be positively correlated with the bed occupancy ratio, implying that an increase in the bed occupancy ratio would result in an increase in the efficiency score. The facilities can invest more to improve the conducive factors that can attract patients, which might improve the utilization of resources and ultimately increase the efficiency of the SDHs [47, 60].

Compared to the Barisal division, the TE score of SDHs was found to be positively associated with division and it was significant for all but the Chattogram division. Barisal is a low-lying, riverine region with many rivers and waterways, and faces challenges related to poor transportation infrastructure, which can affect travel to health facilities in accessing healthcare services. The lower level of efficiency of SDHs in Barisal could be attributed to lower utilization of healthcare facilities due to a lack of readiness to provide healthcare services. A previous study found that the readiness score of health facilities in the Barisal and Sylhet divisions in providing antenatal care services is lower compared to other divisions [61].

The average length of stay and ratio of beds to nurses had a significant negative association with the efficiency score, meaning that a one-unit change in these factors will reduce efficiency by 0.067 and 0.042, respectively. A study conducted in Eritrea also found that the average length of inpatient stay was significantly correlated to hospital inefficiencies [62]. As a longer stay consumes more resources like hospital beds, staff time, and medical equipment, it may lead to inefficiency.

It was evident from the sensitivity analysis that efficiency scores are highly sensitive to the choice of models, with SFA generally producing higher efficiency scores compared to the DEA models. The evidence from other studies is that SFA estimates are generally higher than DEA [63]. This sensitivity analysis provides important insights into the factors influencing TE and highlights the necessity of carefully selecting appropriate models and outputs for robust efficiency evaluations in healthcare settings. Compared to the output-oriented model, the efficiency scores were higher by about 0.08 when using the VRS input-oriented model. However, considering the higher healthcare needs of the current population in Bangladesh, it would not be practical to reduce the input levels and remain at the current level of output. Rather, improving efficiency will be useful for serving more people to meet their healthcare demands. The significant correlations between DEA and SFA efficiency scores suggested a consistent pattern of efficiency estimates, indicating the robustness of the efficiency measurements and the accuracy of our model selection.

### Strengths and limitations

We used multiple approaches for estimating the efficiency of SDHs and compared the overall efficiency scores for robustness of the findings. This is the first study that analysed the TE of the SDHs in Bangladesh to understand the level of efficiency across the SDHs and related determinants. Despite these strengths, this study has several limitations. Firstly, the DEA method does not account for the quality of health services in the production function. However, the level of services provided by SDHs is generally similar, and previous studies have shown the overall quality of healthcare does not vary a lot [64].

Secondly, we could have used other input indicators, such as the price of the drugs. However, as the Central Medical Store Depot of the MOHFW centrally procures drugs, medical supplies, and equipment, the prices are not likely to vary across the SDHs [28], and we did not have access to such data either. This study was based on cross-sectional data. Future research could use data collected at multiple time points and examine how efficiency has changed in the SDHs over time. The potential for endogeneity is another significant limitation of this study, as internal factors within the SDHs under assessment may have an impact on the variables being studied. However, we have used the Simar and Wilson technique in the second stage regression model to estimate the bias-corrected efficiency score and determine the associated factors with efficiency [27].

## Conclusions

The findings of this efficiency study provided empirical evidence on the TE of the sub-district level public PHC facilities in Bangladesh, along with the influence of external and internal factors. A majority of SDHs were technically inefficient in producing healthcare services. The average TE of the SDHs was half of the best score, suggesting there is scope for overall improvement among the inefficient facilities by learning from the efficient facilities, such as improving the human resource structure in the inefficient facilities. Policymakers and hospital managers can use the efficiency estimates of this study to promote benchmarking among the SDHs, where inefficient SDHs can learn from efficient SDHs. Besides, local government can also make use of the results of this study for benchmarking among SDHs. The MOHFW of Bangladesh allocates resources to SDHs based on the number of beds rather than on need assessment. The MOHFW may improve its monitoring system to investigate why some facilities are performing well using similar resources while others are not, and adjust the payment system to take into account the quantity and quality of care.

## Supplementary Information

Supplementary Material 1.

## Data Availability

All data generated or analysed during this study are publicly available and can be accessed at free of costs.

## Appendix 1

### Simar and Wilson bias correction and bootstrapping approach

#### Step 1:

A DEA output-orientated efficiency score $\widehat{{\updelta }_{i}}$ is calculated for each sub-district hospital i using the original data following

$$\mathrm{Max}\;\mathrm\phi\;=\;\overset{\mathrm s}{\underset{\mathrm i=1}{\sum\;{\mathrm A}_{\mathrm r}\;{\mathrm Y}_{\mathrm r0\;}+{\mathrm A}_0}}$$

- $$Subject\, to$$
- $$\sum_{i=1}^{m}{B}_{i} {X}_{{i}_{0}}=1$$ ––––––– (Eq. 1)
- $$\sum_{r=1}^{s}{A}_{r}{Y}_{rj}+{A}_{0}-\sum_{i=1}^{m}{B}_{i} {X}_{ij}\le 0, \forall j=1,\dots ,n$$
- $${A}_{r}{, B}_{i} \ge 0;{A}_{0} is free in sign$$
- where, Y_rj_ = amount of output r from facility j, A_r_ = weight given to output r and $\sum_{r=1}^{s}{A}_{r}{Y}_{rj}$ is the weighted sum of outputs for facility j
- X_ij_ = amount of input i to facility j, B_i_ = weight given to input i, and $\sum_{i=1}^{m}{B}_{i} {X}_{ij}$ is the weighted sum of inputs for facility j
- n = number of facilities.
- s = number of outputs.
- m = number of inputs.
- ${A}_{0}$> 0 defines increasing returns to scale, A_0_ = 0 defines CRS, and A_0_ < 0 defines decreasing returns to scale.

#### Step 2:

Truncated regression is used to model efficiency score $\hat{{\updelta} }_{i}$ as a function of explanatory variables $\left(z_i\right)$ and estimated using maximum likelihood only for the facilities with efficiency score $\hat{{\updelta }}_{i}>1$ where m < n. The estimates are regression coefficient $(\widehat{\upbeta })$ and standard error of the residuals $\left(\hat{\sigma}_\in\right)$

#### Step 3:

For each SDH $\left(i=1\dots..n\right)$, the next three steps (i–iii) are repeated L_1_ times to yield p set of bootstrap efficiency scores $\left(\hat{{\upbeta} }^{*},\hat{{\sigma *}}_{\in }\right)$.

i. For each observation $\left(i=1\dots..n\right)$, draw a random error ${\in }_{i}$ from a truncated normal distribution with mean 0 and standard deviation $\hat{{\sigma }_{\in }}$ with left truncation at $\left(1-{\mathrm z}_{\mathrm i}\;\hat{\mathrm B}\right)$ 
ii. For each observation $\left(i=1\dots ..n\right)$ compute efficiency score $\hat{{\updelta }}^{*}$ = $\left({z}_{i}\widehat{\upbeta }+{\in }_{i}\right)$ and a pseudo data set ${x^\ast}_{i\;=} {x}_{i}$ and ${{y}^{*}}_{i}$ =${y}_{i}{\updelta }_{i}/{{\updelta }}_{i}^{*}$ 
iii. New efficiency score is computed $\hat{{\updelta }}^{*}$ for each observation using the adjusted data.

#### Step 4:

For each firm $\left(i=1\dots ..n\right)$, Use the bootstrap estimates $\hat{{{\updelta }}^{*}_{i}}$ to calculate bias-corrected efficiency scores ​ $\hat{{{\updelta }}^{BC}_{i}}$ = $\hat{{\updelta }}_{i}-{\hat{bias}}_{i}$ where bias is the bootstrap estimator of bias obtained as ${\hat{bias}}_{i}=\left(\frac{1}{L1}\sum_{p=1}^{p}\hat{{\updelta }_{i}}{p}^{*}\right)-\hat{{\updelta }_{i}}$

#### Step 5:

Use the maximum likelihood method to estimate the truncated regression using $\hat{{{\updelta }^{BC}}_{i}}$ on ${z}_{i}$ explanatory variables to obtain updated coefficients $\left(\widehat{\widehat{\upbeta }}\right)$ and standard error $\left(\hat{\hat{{\sigma }_{\in }}}\right)$.

#### Step 6:

Perform another round of bootstrap sampling following three steps​ L_2_ times to refine confidence intervals.

i. For each observation $\left(i=1\dots ..n\right)$ draw a random error ${\in }_{i}$ from a truncated normal distribution with mean 0 and standard deviation $\hat{\hat{{\sigma }_{\in }}}$ with left truncation at $\left(1-{z}_{i}\widehat{\widehat{\upbeta }}\right)$ 
ii. For each observation $$\left(i=1\dots ..n\right)$$ compute efficiency score $${{\updelta }^{**}}_{i}$$ = $$\left({z}_{i}\widehat{\widehat{\upbeta }}+{\in }_{i}\right)$$. 
iii. Use the maximum likelihood method to estimate the truncated regression of $${{\updelta }^{**}}_{i}on {z}_{i}$$ to produce bootstrap estimate $${\hat{\hat{\upbeta }}}^{*}, \hat{\hat{{\sigma }^{*}}}$$ 

#### Step 7:

Use the result to construct confidence interval for $\mathrm\beta\;\mathrm{and}\;{\mathrm\sigma}_{\mathrm\varepsilon}$ 

## Appendix 2

### Translog function for SFA

$$\begin{aligned}\mathrm{ln}\left(Total patient days\right)&={\alpha }_{0}+{\alpha }_{d}lnD+{\alpha }_{n}\mathit{ln} N+{\alpha }_{b}\mathrm{ln}B+{\alpha }_{mt}\mathrm{ln}MT\\&+{\frac{1}{2}\beta }_{dd}(\mathrm{ln}D\times \mathrm{ln}D)+{\frac{1}{2}\beta }_{nn}(\mathrm{ln}N\times \mathrm{ln}N)\\&+{\frac{1}{2}\beta }_{bb}(\mathrm{ln}B\times \mathrm{ln}B)+{\frac{1}{2}\beta }_{mtmt}(\mathrm{ln}MT\times \mathrm{ln}MT)\\&+{\beta }_{dn}(\mathrm{ln}D\times \mathrm{ln}N)+{\beta }_{db}(ln D\times \mathrm{ln}B)\\&+{\beta }_{dmt}(ln D\times \mathrm{ln}MT)+{\beta }_{nb}(ln N\times \mathrm{ln}B)\\&+{\beta }_{nmt}(ln N\times \mathrm{ln}MT)+{\beta }_{bmt}(ln B\times \mathrm{ln}MT)+({v}_{i}-{u}_{i})\end{aligned}$$

## Appendix 3

### Multioutput translog distance function

$$\begin{aligned}\mathrm{ln}\left(OI\right)&={\alpha }_{0}+{\alpha }_{d}lnD+{\alpha }_{n}\mathit{lnN}+{\alpha }_{b}lnB\\&+{\alpha }_{mt}lnMT+{\frac{1}{2}\beta }_{dd}(lnD\times \mathrm{ln}D)+{\frac{1}{2}\beta }_{nn}(ln N\times \mathrm{ln}N)\\&+{\frac{1}{2}\beta }_{bb}(lnB\times \mathrm{ln}B)+{\frac{1}{2}\beta }_{mtmt}(ln MT\times \mathrm{ln}MT)+{\beta }_{dn}(ln D\times \mathrm{ln}N)\\&+{\beta }_{db}(ln D\times \mathrm{ln}B)+{\beta }_{dmt}(lnD\times \mathrm{ln}MT)+{\beta }_{nb}(ln N\times \mathrm{ln}B)\\&+{\beta }_{nmt}(ln N\times \mathrm{ln}MT)+{\beta }_{bmt}(ln B\times \mathrm{ln}MT)+{\beta }_{oi}ln\left(OI\right)\\&+\frac{1}{2}{\beta }_{oioi}(lnOI\times lnOI)+{\beta }_{doi}(ln D\times \mathrm{ln}OI)+{\beta }_{noi}(ln N\times \mathrm{ln}OI)\\&+{\beta }_{boi}(ln B\times \mathrm{ln}OI)+{\beta }_{mtoi}(\mathrm{ln}MT\times \mathrm{ln}OI)+({v}_{i}-{u}_{i}) \end{aligned}$$

where:

D: Number of doctors.

N: Number of nurses.

B: Number of beds.

MT: Number of medical technologists.

OI = ratio or output to input.

Parameters:

- α₀: Constant term (intercept).

- αᵢ: Coefficients representing the direct effects of each input on total patient days.

- βᵢᵢ: Coefficients for the squared logarithmic terms, capturing diminishing or increasing returns to each input.

- βᵢⱼ: Coefficients for the interaction terms, capturing the combined effects of pairs of inputs.

## Abbreviations

AIC: Akaike Information Criterion
BIC: Bayesian Information Criterion
ALoS: Average Length of Stay
BCC: Banker, Charnes, and Cooper
BOR: Bed Occupancy Ratio
CCR: Charnes, Cooper, and Rhodes
CRS: Constant Returns to Scale
DEA: Data Envelopment Analysis
DMU: Decision Making Unit
DGFP: Directorate General of Family Planning
DGHS: Directorate General of Health Services
LMIC: Low-and-Middle Income Countries
MOHFW: Ministry of Health and Family Welfare
OLS: Ordinary Least Squares
PHC: Primary Healthcare
BTN: Ratio of Beds to Nurse
BTP: Ratio of Beds to Physicians
NTP: Ratio of Nurse to Physician
SFA: Stochastic Frontier Analysis
SDH: Sub-District Hospitals
SDG: Sustainable Development Goal
TE: Technical Efficiency
VRS: Variable Returns to Scale
