# Supplementary material for: Technical efficiency of sub-district level hospitals in Bangladesh: a comparative frontier analysis
Source: Health Econ Rev. 2026 Mar 9;16:48. doi: 10.1186/s13561-026-00748-6 (PMC13085694; doi:10.1186/s13561-026-00748-6)
Supplement: Supplementary file 1 — Supplementary Material 1. [file 13561_2026_748_MOESM1_ESM.docx]

**List of Supplementary Tables**

| **ST1: Results from the multioutput distance function in stochastic frontier analysis** | |
| --- | --- |
|  | **Output=Inpatient and outpatient** |
| **Variables** | **Coefficient (95% CI)** |
| Ln (doctor) | 0.568 (-1.833,2.969) |
| Ln (nurse) | 0.333 (-1.049,1.716) |
| Ln (bed) | 0.509 (-1.406,2.423) |
| Ln (MT) | -1.430* (-2.851,-0.009) |
| Ln (doctor) x Ln (doctor) | 0.217 (-0.110,0.543) |
| Ln (nurse) x Ln (nurse) | -0.021 (-0.220,0.178) |
| Ln (bed) x Ln (bed) | -0.125 (-0.592,0.341) |
| Ln (MT) x Ln (MT) | -0.056 (-0.271,0.159) |
| Ln (doctor) x Ln (nurse) | -0.122 (-0.309,0.065) |
| Ln (doctor) x Ln (bed) | -0.235 (-0.718,0.249) |
| Ln (doctor) x Ln (MT) | 0.047 (-0.135,0.230) |
| Ln (nurse) x Ln (bed) | -0.091 (-0.317,0.135) |
| Ln (nurse) x Ln (MT) | 0.120 (-0.051,0.291) |
| Ln (bed) x Ln (MT) | 0.283 (-0.033,0.599) |
| Ln (outpatient/inpatient) | -7.314*** (-9.933,-4.695) |
| Ln (outpatient/inpatient) x Ln (doctor) | 0.222 (-0.525,0.969) |
| Ln (outpatient/inpatient) x Ln (nurse) | 0.152 (-0.416,0.719) |
| Ln (outpatient/inpatient) x Ln (bed) | 0.222 (-0.513,0.957) |
| Ln (outpatient/inpatient) x Ln (MT) | 0.044 (-0.312,0.399) |
| Ln (outpatient/inpatient) x Ln (outpatient/inpatient) | 1.388*** (1.066,1.711) |
| Constant | 12.397*** (5.797,18.998) |
| Constant for mean inefficiency, Mu | -3.199 (-17.808,11.410) |
| Variance of technical inefficiency (ln sigma_u2) | 0.059 (-3.601,3.719) |
| Variance of random error (ln sigma_v2) | -3.545*** (-4.135,-2.954) |
| Sd of technical inefficiency (sigma_u) | 1.03 (0.165-6.419) |
| Sd of random error (sigma_v) | 0.17 (0.126-0.228) |
| Ration of sigma_u / sigma_v | 6.06 (4.206-7.915) |
| Loglikelihood | -83.576 |
| AIC | 215.152 |
| BIC | 312.289 |
| ** =p<0.05; ** =p<0.01; *** =p<0.001* |  |

| **ST2: Results from the Cobb-Douglas stochastic frontier analysis** | |
| --- | --- |
|  | **Output=Total patient days** |
| **Variables** | **Coefficient (95% CI)** |
| Ln (doctor) | 0.358*** (0.26, 0.45) |
| Ln (nurse) | 0.050 (-0.05, 0.15) |
| Ln (bed) | 0.064 (-0.11, 0.24) |
| Ln (MT) | 0.133** (0.05, 0.22) |
| Constant | 7.309*** (6.60, 8.02) |
| Constant for mean inefficiency, Mu | -513.065*** (-3634.66, 2608.54) |
| Variance of technical inefficiency (ln sigma_u2) | 5.232*** (-0.842, 11.307) |
| Variance of random error (ln sigma_v2) | -2.819 (-3.17, -2.47) |
| Sd of technical inefficiency (sigma_u) | 13.682*** (0.66, 285.26) |
| Sd of random error (sigma_v) | 0.244*** (0.21, 0.29) |
| Ratio of sigma_u / sigma_v | 56.010 (14.45, 97.57) |
| Loglikelihood | -217.729 |
| AIC | 451.457 |
| BIC | 483.836 |
|  |  |

| **ST3: Results from the translog stochastic frontier analysis** | |
| --- | --- |
|  | **Output=Total patient days** |
| **Variables** | **Coefficient (95% CI)** |
| Ln (doctor) | 2.533** (0.67, 4.39) |
| Ln (nurse) | 1.070 (-0.07, 2.21) |
| Ln (bed) | 1.702* (-0.04, 3.44) |
| Ln (MT) | -2.065** (-3.63, -0.49) |
| Ln (doctor) x Ln (doctor) | 0.394 (-0.02, 0.81) |
| Ln (nurse) x Ln (nurse) | -0.045 (-0.30, 0.21) |
| Ln (bed) x Ln (bed) | -0.051 (-0.65, 0.55) |
| Ln (MT) x Ln (MT) | -0.101 (-0.35, 0.15) |
| Ln (doctor) x Ln (nurse) | -0.219 (-0.45, 0.02) |
| Ln (doctor) x Ln (bed) | -0.717** (-1.26, -0.18) |
| Ln (doctor) x Ln (MT) | 0.121 (-0.11, 0.35) |
| Ln (nurse) x Ln (bed) | -0.157 (-0.45, 0.13) |
| Ln (nurse) x Ln (MT) | 0.166 (-0.04, 0.37) |
| Ln (bed) x Ln (MT) | 0.411* (0.01, 0.82) |
| Constant | 2.020 (-2.83, 6.87) |
| Constant for mean inefficiency, Mu | -475.652 (-3,059.40, 2,108.10) |
| Variance of technical inefficiency (ln sigma_u2) | 5.082 (-0.34, 10.51) |
| Variance of random error (ln sigma_v2) | -2.888*** (-3.25, -2.53) |
| Sd of technical inefficiency (sigma_u) | 12.692 (0.84, 191.10) |
| Sd of random error (sigma_v) | 0.236*** (0.20, 0.28) |
| Ratio of sigma_u / sigma_v | 53.788** (19.37, 88.21) |
| Loglikelihood | -193.795 |
| AIC | 423.59 |
| BIC | 496.443 |
| ** =p<0.05; ** =p<0.01; *** =p<0.001* |  |
